# Are trace element concentrations suitable biomarkers for the diagnosis of cancer?

**DOI:** 10.1016/j.redox.2021.101900

**Published:** 2021-02-18

**Authors:** Kristina Lossow, Maria Schwarz, Anna P. Kipp

**Affiliations:** a Department of Molecular Nutritional Physiology, Institute of Nutritional Sciences, Friedrich Schiller University Jena, Jena, 07743, Germany; b TraceAge-DFG Research Unit on Interactions of Essential Trace Elements in Healthy and Diseased Elderly, Potsdam-Berlin-Jena-Wuppertal, Germany

**Keywords:** Cancer, Selenium, Iron, Copper, Zinc

## Abstract

Despite advances in cancer research, cancer is still one of the leading causes of death worldwide. An early diagnosis substantially increases the survival rate and treatment success. Thus, it is important to establish biomarkers which could reliably identify cancer patients. As cancer is associated with changes in the systemic trace element status and distribution, serum concentrations of selenium, iron, copper, and zinc could contribute to an early diagnosis. To test this hypothesis, case control studies measuring trace elements in cancer patients vs. matched controls were selected and discussed focusing on lung, prostate, breast, and colorectal cancer. Overall, cancer patients had elevated serum copper and diminished zinc levels, while selenium and iron did not show consistent changes for all four cancer types. Within the tumor tissue, mainly copper and selenium are accumulating. Whether these concentrations also predict the survival probability of cancer patients needs to be further investigated.

## Abbreviations

ATP7a: ATPase copper transporting alpha
BC: breast cancer
CRC: colorectal cancer
Cu: copper
Ctr1: Cu transporter 1
EPIC: European Prospective Investigation into Cancer and Nutrition
Fe: iron
GPX(s): glutathione peroxidase(s)
HCC: hepatocellular carcinoma
LC: lung cancer
OR: odds ratio
PC: prostate cancer
RR: relative risk
Se: selenium
SELENOP: selenoprotein P
SEPHS2: selenophosphate synthase 2
SLC7A11: solute carrier family 7 member 11
SMD: standardized mean difference
TAM: tumor-associated macrophages
Zn: zinc
TE(s): trace element(s)
TIBC: total Fe binding capacity
TSAT: transferrin saturation

## 1 Introduction

Cancer is a multifactorial disease causing 9.6 million deaths annually [1]. Both sexes combined, the most common organs susceptible to cancer development are lung (LC), breast (BC), prostate (PC), and colon/rectum (CRC) [1]. Cancer is characterized by a progressive transformation of healthy cells into malignant progeny. During this process, tumor cells acquire novel properties over time resulting in e.g., unrestricted proliferation and invasion capacity which have been summarized in the so-called ‘hallmarks of cancer’ [2]. In general, progression of cancer is expressed as cancer stages, reflecting the spread of tumors from unspread (I) to growing tumors penetrating in the adjacent tissues (II-III) or other organs (IV). Depending on the tumor type, these different stages can be identified and characterized by changes of tumor biomarkers, e.g., K-Ras, HER2/NEU or different cancer antigens [3,4]. As early diagnosis is one of the most important contributors to a successful cancer therapy, there is an urgent need for further reliable biomarkers for detection of malignant cells in the body. During the last decades, the focus was mainly on the identification of mutations in driver genes which were both used for diagnostics but also for a personalized tumor therapy [5]. Besides genetic modifications, tumor cells are characterized by substantial changes in their metabolism affecting the need for macronutrients but also for micronutrients. Already in 1975, Schwartz reviewed the role of trace elements (TEs) including copper (Cu), selenium (Se), and zinc (Zn) in cancer, discussing their potential roles as diagnostic or prognostic markers [6]. In this mini review, we aim to extend the current view by further testing the hypothesis whether concentrations of the TEs Se, iron (Fe), Cu, and Zn are suitable predictors for the diagnosis of cancer. To do so, recently published case-control studies of the most common cancer types that determined TE concentrations in serum/plasma or within the tumor tissue were selected. As the measurement of total TE concentrations might not appropriately indicate functional impairments, as reported e.g., for Zn [7], also biomarkers are used for describing the TE status [8]. While selenoproteins such as selenoprotein P (SELENOP) and plasma glutathione peroxidase (GPX3) are frequently used to analyse the Se status, the Cu status can additionally be described by ceruloplasmin concentrations. For Zn, the amount of free Zn in serum is discussed as more appropriate biomarker but this still needs to be validated further. Next to the Fe concentration itself, the Fe storage and transport proteins ferritin and transferrin, respectively, are used to assess the Fe status. Further biomarkers include total Fe binding capacity (TIBC), indicating the capacity of transferrin to bind Fe, and transferrin saturation (TSAT), calculated by dividing serum Fe by TIBC [9]. However, several of these biomarkers are frequently distorted by other clinical factors e.g., inflammation, hydration status or hemolysis (in case of serum Fe), resulting in incorrect conclusions regarding the patient's TE status [9,10]. The physiological reference ranges for the considered TEs are given in Table 1.

**Table 1:** Reference ranges for the trace elements selenium (Se), iron (Fe), copper (Cu), and zinc (Zn).

Trace element	Reference ranges in serum	Reference
Se	70-150 μg/l	[11]
Fe	men: 0.55–1.60 mg/l women: 0.40–1.55 mg/l	[12]
Cu	0.64–1.40 mg/l	[12]
Zn	0.66–1.10 mg/l	[13]

## 2 Selenium

Se becomes cotranslationally incorporated into selenoproteins, encoded by 25 genes in humans. Most selenoproteins like GPXs or thioredoxin reductases are important regulators of cellular redox balance [14]. With regard to primary tumor prevention, the antioxidant function of selenoproteins appears to protect from DNA damage and thus tumor initiation. Several large prospective studies, including the European Prospective Investigation into Cancer and Nutrition (EPIC) study with more than 521,000 study participants enrolled in 10 western European countries, have shown that a low Se status increases the risk to develop cancer e.g., CRC [15]. Likewise, a large meta-analysis revealed a protective effect of higher Se serum/plasma concentration compared to the lowest category for all observed cancer types [16]. On the contrary, others conclude from the available data that there is no evidence for overall cancer prevention by a higher Se intake [17].

Recent case-control studies with BC patients revealed a consistent picture regarding the relationship between serum Se levels and cancer. The comparison of 229 BC patients and 200 healthy controls in two Korean studies indicated a significant reduction of Se concentrations in the blood of BC patients (107 vs. 109 μg/l, p = 0.024) or at least its trend (98 vs. 102 μg/l, p = 0.085) [18]. Comparably, two Indian studies with 140 BC and 140 healthy patients reported significantly diminished blood Se levels in cancer patients compared to controls (47 vs. 102 μg/l^1^, p < 0.0001 [19]; 43 vs. 68 μg/l, p < 0.05 [20]). The changes in serum Se concentrations might also affect the Se content of tumor tissue. The analysis of 42 sets of tumors and adjacent healthy tissue revealed significantly higher Se concentrations in BC tumors as compared to adjacent tissue (0.15 vs. 0.05 μg/g, p < 0.0001) [21]. This observation was already described a decade ago, with almost four-times higher Se levels in neoplastic breast tissue compared to healthy surrounding tissue [22], indicating a redistribution of Se.

For the second most prevalent cancer in males, PC, a recent study comparing serum samples of 141 cases and 114 controls within the Singapore Prostate Cancer Study reported 1.2-times higher Se concentrations among cancer cases in comparison to controls (131 vs. 109 μg/l, p < 0.0001) [23]. Contrary, analysis of blood Se in PC patients (n = 20) compared to matched controls (n = 21) from Turkey showed lower values in PC patients (77 vs. 95 μg/l, p < 0.001) [24]. In prostatic tissue, no variances between 36 patients with adenocarcinoma and 37 healthy males were recognized for Se values (0.6 vs. 0.8 μg/g, p = 0.16) [25].

Similarly, when 440 incident LC cases and matched 1,320 healthy controls from the Dongfeng-Tongji Cohort (China) were compared, both, cancer and control cases, showed comparable Se levels in plasma (60 vs. 59 μg/l, p = 0.551) [26]. SELENOP is supposed to be a more suitable marker to reflect the Se status. A study including serum samples from 48 mostly male LC patients and 39 healthy controls not only quantified SELENOP but further selenoproteins and selenometabolites. The sum of selenocompounds tended to be higher in LC patients than in controls (171 vs. 149 μg/l^2^, p > 0.05) [27].

A case-control subgroup of the EPIC cohort was analyzed regarding pre-diagnostic Se status before development of CRC, including 966 individuals who were diagnosed for cancer in the follow-up and 966 matched controls. Whereas serum Se concentration did not differ between cases and controls in total (p_crc_ = 0.147) and among cancer sub-sites (colon 80 vs. 82 μg/l, p_colon_ = 0.097; rectum 83 vs. 84 μg/l, p_rectum_ = 0.816), SELENOP concentrations were reduced in cases compared to controls in total (4200 vs. 4300 μg/l, p = 0.027) and in colon cancer (4100 vs. 4300 μg/l, p = 0.008) [15]. In line with cancer-driven changes in breast tumor tissue, the Se content of malignant colon samples (n = 59) were significantly higher than in adjacent healthy tissue (0.17 vs. 0.11 μg/g, p=<0.0001) [28].

Overall, the data show that Se homeostasis is modulated in cancer patients. While plasma/serum Se levels were reduced in BC, no consistent changes were observed for LC, PC, and CRC patients. However, Se concentrations increased in tumor tissue of BC and CRC. Recently, a mechanism has been proposed explaining accumulation of Se in tumor tissue [29]. Especially breast but also other cancer cells are described to be selenophilic, catalyzed by the cystine/glutamate antiporter solute carrier family 7 member 11 (SLC7A11) that promotes Se uptake and selenoprotein synthesis. In addition, selenophosphate synthase 2 (SEPHS2) essential for selenoprotein synthesis was upregulated in BC compared with normal breast tissue, positively associated with poor survival of BC patients [30]. Thus, several types of tumors actively accumulate Se. Whether this is also the reason for lower systemic Se levels is unclear so far.

## 3 Iron

Fe is essential for basal cellular processes including utilization of oxygen or DNA synthesis [31]. However, high Fe concentrations are detrimental for cells resulting in concomitant lipid peroxidation and cell death via ferroptosis [32]. But eventually not all cells die in response to excess Fe levels as Fe overload favors tumor development which has been shown e.g., for hepatocellular carcinoma (HCC) and other types of cancer [33,34].

A case-cohort study, based on the EPIC-Heidelberg cohort, analyzed pre-diagnostic serum concentrations of Fe, ferritin, transferrin, and TSAT in relation to cancer risk and mortality in a random subcohort (n = 2,738) and incident cases of CRC (n = 256), LC (n = 195), BC (n = 627), PC (n = 554), and cancer mortality (n = 759) 10 years after basal evaluation [35]. Here, none of the analyzed markers was significantly associated with PC, LC or CRC risk. Only ferritin levels were inversely associated with BC risk (HR = 0.67, p = 0.04) and total cancer mortality (HR = 0.70, p = 0.02). In line with this, the ‘Rotterdam Study’ with 5,435 participants and a follow-up period of 22 years reported an inverse correlation between Fe intake and LC risk (HR = 0.58, p = 0.021) [36]. On the contrary, a meta-analysis revealed an increased risk for CRC (RR = 1.14) with higher heme Fe intake based on eight prospective cohort studies [37].

There is not much evidence of possible shifts in blood Fe levels based on case-control studies with regard to BC. An Indian study with 40 healthy and 40 BC patients’ blood samples showed significantly higher Fe levels in cancer patients in comparison to controls (3.38 vs. 1.68 mg/l*, p < 0.0001) [19]. Also, tissue sets (tumor and adjacent tissues) from 42 women diagnosed with primary BC in Poland revealed significantly higher contents of Fe in tumors compared to adjacent tissue (67 vs. 41 μg/g, p = 0.044) [21].

There is also very little data on PC, especially with regard to studies from the past decade. However, one study compared blood Fe level of PC patients (n = 74) with matched healthy controls (n = 66), revealing higher Fe concentrations in cancer patients (902 vs. 492 mg/l^2^, p < 0.05) [38]. Older studies do not indicate altered Fe concentrations in blood and serum from PC patients compared to matched controls (623 vs. 602 mg/l, p > 0.05 [24]; 0.94 vs. 1.03 mg/l^3^, p = 0.278 [39]). However, the latter reported significantly reduced levels for serum ferritin (0.16 vs. 0.255 mg/l, p = 0.043) and TSAT (24 vs. 32%, p = 0.014), and an inverse correlations for TIBC (3.90 vs. 3.37 mg/l^3^, p = 0.018) in cancer cases [39]. A study on Fe levels in prostatic tissue of patients with prostate adenocarcinoma (n = 36) and healthy males (n = 37) showed that the Fe content is higher in PC patients (163 vs. 111 μg/g, p = 0.03) [25].

With regard to LC a recent study from Poland consisting of 200 untreated patients diagnosed for LC and 200 matched controls reported that serum Fe levels (1.40 vs. 1.20 mg/l, p = 0.01), ferritin (0.26 vs. 0.22 μg/l, p = 0.007), and TIBC (3.40 vs. 3.17 mg/l, p = 0.006) were significantly higher in cancer patients compared to healthy controls [40]. Within a meta-analysis with 1,118 LC patients and 832 controls Fe serum levels of cancer patients were unchanged to those of healthy controls (SMD = −0.125, p = 0.189) [41].

CRC is generally assumed to be associated with Fe deficiency, which is claimed to be prevalent in approximately 60% of patients, probably due to chronic tumor-induced blood loss and reduced intestinal Fe absorption [42]. However, comparing serological samples of 356 cases and 396 controls in the United States did not indicate any differences for Fe (1.07 vs. 1.06 mg/l, p = 0.93), ferritin (0.14 vs. 0.16 mg/l, p = 0.53) or TSAT (31 vs. 30%, p = 0.25). In comparison, TIBC indicate a trend towards higher levels in controls (3.47 vs. 3.51 mg/l, p = 0.07) [43]. An Iranian study reported significantly diminished Fe concentrations (CRC 8.3x lower, p_crc_ <0.001; colon 4.8x lower, p_colon_ = 0.072; rectum 65.5x lower, p_rectum_ = 0.001) in cancerous tissue in comparison to non-cancerous tissue of the colon and rectum of 50 patients [44]. In contrast, a Serbian analysis of tumor and adjacent healthy tissue in 59 subjects revealed no variances in Fe concentrations (23 vs. 22 μg/g, p = 0.546) [28].

In summary, the cancer-related effects of Fe are not clear yet and might differ with regard to tissue type and cancer progression. Indeed, several studies explained changes in Fe concentrations by altered expression levels of Fe-regulatory proteins/Fe-related genes involved in import, export, and storage of cellular Fe, e.g., in BC pathogenesis (reviewed in Ref. [45]) and liver cancer [46,47]. Additionally, tumor-associated macrophages (TAM) are supposed to affect dysregulation of cancer cells’ Fe metabolism and their microenvironment by providing tumor cells with Fe [48], crucial to fulfill the enhanced demands of Fe for division, growth, and survival.

## 4 Copper

Cu is a redox active metal but Cu-dependent enzymes also contribute to many physiological processes, e.g., cellular respiration, Fe homeostasis, and angiogenesis [49]. Cu is most widely discussed to accumulate in serum and tumor tissue of cancer patients (reviewed in Ref. [50]), but prospective studies are inconsistent regarding a possible link between high Cu levels and an increased cancer risk. While such an association has been described for CRC [51], it has not been reported for HCC and LC [26,36,52].

A recently published case-control study with two independent cohorts from Korea, including 229 BC patients and 200 controls revealed contradicting results. The first cohort showed significantly higher serum Cu concentrations in cancer patients (1.00 vs. 0.95 mg/l, p = 0.0002) with highest values in sera of women with cancer stage IV (p = 0.048), whereas the second cohort did not (0.90 vs. 0.92 mg/l, p = 0.281) [18]. An Indian study including 100 BC patients and 140 healthy controls reported higher Cu serum levels in BC patients compared to healthy controls (1.17 vs. 0.89 mg/l*, p < 0.0005 [19]).

A meta-analysis based on 11 publications with 653 cases and 614 controls, which focused on serum Cu levels, found no significant difference between cancer patients and healthy controls (SMD = 0.01, p = 0.975) [53]. In contrast, a comprehensive meta-analysis that took into account other publications in addition to those of Jouybari et al. [53], ending up by 36 studies with 5,747 female subjects (2,369 BC patients, 901 patients with benign breast diseases, and 2,477 healthy controls) indicated significantly higher serum Cu levels in BC patients than in healthy controls (SMD = 1.99, p < 0.001) and patients with benign breast diseases (SMD = 0.99, p = 0.002) [54].

With regard to BC tissue, an additional meta-analysis compared six studies with regard to Cu levels in breast tissue (129 cases and 156 controls), indicating no differences between BC cases and controls [53]. But there are older studies indicating higher concentrations of Cu in malignant breast tissue [55,56].

A recently published Asian study on PC, comparing PC diagnosed patients (n = 141) with matched controls (n = 114), did not demonstrate variances in serum Cu levels (1.05 vs. 1.05 mg/l, p = 0.37) [23]. Likewise, a study from Pakistan with 74 and 66 PC patients and controls, respectively, showed no difference in blood Cu concentration (2.02 vs. 2.07 mg/l^2^, p > 0.05) [38], whereas another investigation from Turkey revealed higher blood Cu levels in PC patients (n = 20) in relation to matched controls (n = 21) (0.45 vs. 0.28 mg/l, p < 0.0001) [24].

In comparison to PC, the latest studies on LC provide more consistent effects. A comprehensive meta-analysis based on 33 articles including 3,026 LC cases and 9,439 controls identified an association between serum Cu levels and LC. The overall results showed higher serum Cu levels in LC patients compared to controls (SMD = 1.10, p < 0.001) [57]. In accordance with this, a Polish study with blood samples from 44 LC patients and 44 control subjects reported significantly higher serum (but not whole blood) Cu levels (1.32 vs. 1.10 mg/l, p < 0.001) in cancer patients compared to controls. Further, advanced disease had significantly higher whole blood (but not serum) Cu levels (1.19 vs. 0.81 mg/l, p < 0.05) compared to patients with lower clinical stages [58]. The same trend was recognized in a nested case-control study (0.95 vs. 0.90 mg/l, p = 0.044) with 440 incident LC cases and 1,320 matched healthy controls from the Dongfeng-Tongji Cohort (China) [26].

Looking at the distal parts of the alimentary tract, a case-control study nested within the EPIC cohort with 966 CRC cases (569 colon and 370 rectal cancers) and 966 matched controls was considered. Here, CRC cases diagnosed within the first two years after recruitment did reveal markedly increased circulating Cu concentrations (p = 0.005), whereas no association was found for cancer cases diagnosed after more than two years of follow-up (p = 0.990) [51]. In line with this, studies examining the Cu content in CRC tissue observed significantly higher Cu levels than those in control tissue (1.3x higher, p = 0.002 [44]; 1.47 vs. 1.26 μg/g, p = 0.011) [28].

Overall, Cu levels are increased in serum/blood of cancer patients as well as in tumor tissue. This is in line with an increased need of the tumor for Cu to enable angiogenesis, growth, and metastasis [50]. The upregulation of both circulating and intra-tumoral Cu concentrations indicates that the systemic Cu homeostasis is modulated in cancer patients. Tumor cells have been described to accumulate Cu by upregulating Cu transporter 1 (Ctr1) and via macropinocytosis. Additionally, the Cu exporter ATPase copper transporting alpha (ATP7A) is translocated to the plasma membrane to fine tune cellular Cu levels and to protect cells from excessive Cu and concomitant toxicity [59]. Cu levels have been discussed to correlate with poor prognosis and therapy resistance indicating that it could indeed act as a potential marker [50]. Based on this, reducing Cu levels e.g., by treatment with Cu chelators should be further studied as putative therapeutic intervention for cancer patients.

## 5 Zinc

Zn acts as constituent and cofactor of numerous enzymes, involved in signaling pathways important for e.g., proliferation, differentiation, apoptosis, cell cycle regulation, and immune function (reviewed in Refs. [60,61]). Thus, many hallmarks of cancer are affected by Zn. However, a recent cross-sectional study including 3,607 participants did not reveal any association between serum Zn levels and the incidence of (all kind of) cancer in multivariate logistic regression analysis (OR = 1.001, p = 0.980) [62]. Contrary, many other studies indicate an inverse relationship between Zn intake/levels and cancer risk of various tissue types, e.g., BC [48], LC [26,36], CRC [37,51], and HCC [52].

Two recent Korean case-control studies, comparing 229 BC patients with 200 controls in total, revealed either that cancer patients had significantly lower serum Zn levels than controls (0.95 vs. 1.10 mg/l, p < 0.0001) or showed no difference between both groups at all (0.76 vs. 0.75 mg/l, p = 0.596) [18]. Even two times lower Zn concentrations in whole blood samples were observed in BC patients from India comparing 40 BC and 40 healthy patients (0.59 vs. 1.15 mg/l*, p < 0.0001) [19]. The same trend, albeit with only a 1.15-fold lower Zn content in cancer patients, was found in a second Indian study (p < 0.05; n = 100) [20]. A meta-analysis from 2015, including 14 studies with 662 BC patients and 775 healthy controls, did not identify differences of the Zn status between BC patients and healthy subjects (SMD = −0.65) [63]. However, a later analysis based on studies included in Wu et al. and additional publications (from e.g., India and China ending up in 19 summarized studies) indicated that BC patients had significantly lower serum/plasma Zn concentrations than healthy controls (SMD = −1.61, p < 0.001) [64]. The same conclusion was drawn in a recently published meta-analysis, comparing 35 publications primarily from China and India (SMD = −1.20, p < 0.001) [54].

In terms of tissue, several studies indicate that Zn levels in BC tissue are significantly elevated compared with normal tissue, as summarized and verified by Riesop et al. and Rusch et al. [65,66]. Here, 8 and 26 tissue samples of BC cells and healthy stroma, respectively, were analyzed by laser ablation inductively coupled plasma mass spectrometry, revealing about twice as high Zn concentrations in BC tissue (4.2–8.1 vs. 6.9–17.9 mg/kg [65]; 0.8–11.4 vs. 3.5–19.5 mg/kg [66]. Additionally, results indicate a direct correlation between Zn concentration and histological grade [65,66].

Zn is an important factor for prostatic function [67]. Previous observations suggest that related to the development and progression of PC Zn concentrations decline in cancerous tissue [68]. Indeed, lower Zn concentrations were observed in adenocarcinoma of the prostate vs. healthy tissue (n = 36 vs. 37; 122 vs. 1,031 μg/g, p < 0.0001) [25]. However, inconsistent results are reported with regard to serum Zn levels (reviewed in Ref. [69]). Within the Singapore Prostate Cancer Study mean Zn concentrations in serum samples of 141 cases were 1.2 times higher (0.86 vs. 0.69 mg/l, p < 0.0001) than in 114 control samples [23]. A study consisting of 197 patients and 197 healthy participants, reported a less pronounced increase of serum Zn levels in PC patients (0.90 vs. 0.86 mg/l, p < 0.1) [70]. Contradictory, the comparison of 220 PC patients and 220 age-matched healthy controls revealed lower plasma Zn levels (0.62 vs. 1.00 mg/l^3^, p < 0.001) in the PC patients and even further reduced Zn concentrations in males with a higher disease grade [71]. Congruently, within a comprehensive meta-analysis including 1,318 patients with prostate disease and 1,413 controls serum Zn concentrations of PC patients were significantly lower compared to healthy controls (SMD = −0.94) [72]. Two other studies also indicated lower blood Zn values in PC patients compared to matched controls (4.4 vs. 5.8 mg/l, p < 0.001, [24]; 4.54 vs. 7.00 mg/l^2^, p < 0.05 [38]).

Comparable trends were reported in a current meta-analysis with respect to LC [73]. Here, 27 from 32 studies (including 2,894 cases and 9,419 controls), mostly from Asia (including three European studies), indicated lower serum Zn levels in LC patients than in controls (SMD = −0.88, p < 0.001), while in four and two studies no significant and a positive association, respectively, were recognized. In a further nested case-control study 440 incident LC cases and 1,320 matched healthy controls were compared, revealing higher Zn levels in controls (1.18 vs. 1.28 mg/l, p = 0.019) [26].

For the colon, a nested case-control study was conducted within the EPIC cohort to investigate the association between serum Zn concentrations with the risk to develop CRC. Therefore, Zn levels of 966 cases (569 colon and 370 rectal cancers) and 966 matched controls were determined, revealing no difference (0.96 vs. 0.97 mg/l, p = 0.37) [51]. The same was true for cancerous rectum tissue (mean difference 1.2x, p = 0.379) from 50 CRC patients, whereas colon tissue revealed higher Zn concentrations than adjacent healthy tissue (0.12 vs. 0.00 μg/ml, p = 0.001) [44]. In contrast, 59 malignant colon (mainly CRC stage III) and adjacent healthy tissue samples from CRC patients revealed that the Zn content of tumors was significantly reduced compared to healthy tissue (17 vs. 19 mg/kg, p < 0.0001) [28].

In summary, blood/serum Zn levels are reduced in most cancer patients, which has been linked to an aberrant expression of Zn transporters, e.g., in breast and prostate cancer (reviewed in Refs. [[74], [75], [76]]), indicating that comparable to Cu homeostasis systemic Zn homeostasis is also affected by cancer independent of the tissue of origin. However, Zn concentrations within the malignant tissue is more variable and appears to depend on the affected tissue and probably the stage and malignancy of the respective tumor.

## 6 Perspective

The studies presented here describe the current state of knowledge on TE concentrations in the serum and tumor tissue of cancer patients. On the one hand, TE disturbances may be involved in tumor initiation by increasing cell damage, DNA injuries, and cellular redox imbalance. On the other hand, redistribution of TEs may be a feature which develops during tumorigenesis. Based on the available data it is very difficult to judge whether changes in TE homeostasis are one of the factors causing tumor development or whether they are just a consequence of malignant transformation. In blood, Cu concentrations appear to be increased while Zn levels decrease in cancer patients. As for Cu consistent changes in circulating blood and in tumor tissue occur, principles of homeostatic regulation might be deregulated in cancer patients. In contrast, Se levels are specifically accumulating within tumor tissue while serum/plasma Se levels are rather constant or even declining in cancer patients. For Fe the picture is rather heterogeneous and changes obviously depend on the type of cancer, as it’s the case for Zn concentrations in cancer tissue.

**Fig. 1:**
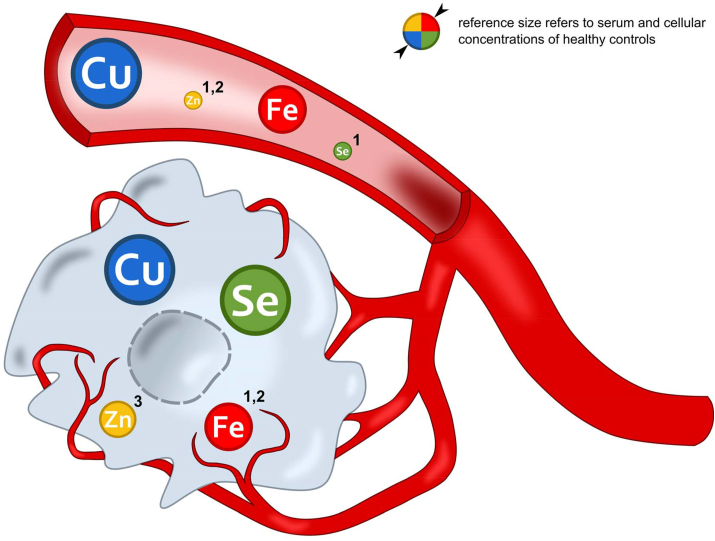
Shifting trace element concentrations including selenium (Se), iron (Fe), copper (Cu), and zinc (Zn) in serum (depicted as blood vessel) and tumor tissue (depicted as cell) of cancer patients based on case-control studies published within the last years and referred within this review. Trace element differences of cancer patients compared to controls are illustrated by size deviations from the reference circle. ^1^ particularly robust in breast cancer studies, ^2^ particularly robust in prostate cancer, ^3^ depending on cancer type contradictory changes occur.

Herein, we describe TE profiles for four TEs which appear to be typical for cancer (Fig. 1). However, these profiles are based on different studies and the comparison of the data is very limited due to study differences in geographic, gender, age, sample size, sample collection, and (at least partly) missing classification of tumor grade. Some of the studies reported TE concentrations far beyond physiological concentrations (Table 1) which might depend on the methods of measurements and sample preparation. Accordingly, it is difficult to conclude whether the reported changes of individual TEs can also be verified within one study population. So far, it cannot be ruled out that the amount of biologically available free TEs is different in tumor tissue in comparison to healthy tissue even though no difference in total TE concentrations could be detected. For future studies, several TEs and functional biomarkers should be analyzed in parallel in whole sample sets to reliably identify TE profiles both in serum and tumor tissue. Extensive verification of the control group should also be performed as the absence of cancer or other diseases is not necessarily synonymous for a healthy control. In this respect, observed differences within the control groups may be subject to bias.

Further, mechanistic studies are needed to understand how tumor cells modulate their TE balance in comparison to healthy cells and how several TEs interfere with each other when shifting TE patterns are achieved. Considering several TEs at once is also very important when modulation of TE levels becomes a therapeutic option. The use of chelators, e.g., to reduce Cu levels, most often also affects other TEs which needs to be taken into account.

So far, available data does not allow to use TE concentrations as biomarker for cancer diagnosis maybe with the exception of Cu which is upregulated very consistently and independent of the type of tumor.

There are a few studies addressing the question whether TE concentrations can predict the survival probability of cancer patients or whether they could even be used as therapeutic targets. To come to this point, more studies are needed to understand underlying mechanisms and to better describe differences in TEs e.g., in relation to tumor stage and other characteristics of a tumor. TEs are essential nutrients but eventually they need to be depleted from tumor tissue in case of Fe, Cu, and Se or even brought back in case of Zn to reduce the survival of cancer cells.

## Funding

This work was supported by the German Research Foundation (DFG) [FOR 2558].

## Declaration of competing interest

The authors declare that they have no known competing financial interests or personal relationships that could have appeared to influence the work reported in this paper.

## Footnotes

1 Recalculated based on published information.

2 Recalculated based on standard serum or whole blood density of 1.03 mg/l and 1.06 mg/l, respectively.

3 Recalculated with molar mass.

## References

[1] Bray F. (2018). Global cancer statistics 2018: GLOBOCAN estimates of incidence and mortality worldwide for 36 cancers in 185 countries. CA A Cancer J. Clin..

[2] Hanahan D., Weinberg R.A. (2011). Hallmarks of cancer: the next generation. Cell.

[3] Nikolouzakis T.K. (2018). Improving diagnosis, prognosis and prediction by using biomarkers in CRC patients (Review). Oncol. Rep..

[4] Ludwig J.A., Weinstein J.N. (2005). Biomarkers in cancer staging, prognosis and treatment selection. Nat. Rev. Canc..

[5] Tokheim C.J. (2016). Evaluating the evaluation of cancer driver genes. Proc. Natl. Acad. Sci. U. S. A..

[6] Schwartz M.K. (1975). Role of trace elements in cancer. Canc. Res..

[7] Bao B. (2010). Zinc decreases C-reactive protein, lipid peroxidation, and inflammatory cytokines in elderly subjects: a potential implication of zinc as an atheroprotective agent. Am. J. Clin. Nutr..

[8] Bornhorst J. (2018). The crux of inept biomarkers for risks and benefits of trace elements. Trac. Trends Anal. Chem..

[9] Pfeiffer C.M., Looker A.C. (2017). Laboratory methodologies for indicators of iron status: strengths, limitations, and analytical challenges. Am. J. Clin. Nutr..

[10] Agarwal A. (2018). Serum zinc levels as a predictor of severity of acute diarrhea. Indian J. Pediatr..

[11] Mayo Foundation for Medical Education and Research SES-Clinical: selenium, s. https://www.mayocliniclabs.com/test-catalog/Clinical+and+Interpretive/9765.

[12] Baudry J. (2020). Changes of trace element status during aging: results of the EPIC-Potsdam cohort study. Eur. J. Nutr..

[13] Mayo Foundation for Medical Education and Research ZNSClinical: zinc, s. https://www.mayocliniclabs.com/test-catalog/Clinical+and+Interpretive/8620.

[14] Labunskyy V.M., Hatfield D.L., Gladyshev V.N. (2014). Selenoproteins: molecular pathways and physiological roles. Physiol. Rev..

[15] Hughes D.J. (2015). Selenium status is associated with colorectal cancer risk in the European prospective investigation of cancer and nutrition cohort. Int. J. Canc..

[16] Cai X. (2016). Selenium exposure and cancer risk: an updated meta-analysis and meta-regression. Sci. Rep..

[17] Vinceti M. (2014). Selenium for preventing cancer. Cochrane Database Syst. Rev..

[18] Choi R. (2018). Serum trace elements and their associations with breast cancer subgroups in Korean breast cancer patients. Nutrients.

[19] Naidu B.G. (2019). Multivariate analysis of trace elemental data obtained from blood serum of breast cancer patients using SRXRF. Results Phys..

[20] Hassan T. (2017). Study of serum levels of trace elements (selenium, copper, zinc, and iron) in breast cancer patients. Int. J. Clin. Oncol. Canc. Res..

[21] Jablonska E. (2017). Cadmium, arsenic, selenium and iron- Implications for tumor progression in breast cancer. Environ. Toxicol. Pharmacol..

[22] Charalabopoulos K. (2006). Selenium in serum and neoplastic tissue in breast cancer: correlation with CEA. Br. J. Canc..

[23] Lim J.T. (2019). Association between serum heavy metals and prostate cancer risk–A multiple metal analysis. Environ. Int..

[24] Ozmen H. (2006). Comparison of the concentration of trace metals (Ni, Zn, Co, Cu and Se), Fe, vitamins A, C and E, and lipid peroxidation in patients with prostate cancer. Clin. Chem. Lab. Med..

[25] Zaichick V., Zaichick S. (2016). Prostatic tissue levels of 43 trace elements in patients with prostate adenocarcinoma. Canc. Clin. Oncol..

[26] Bai Y. (2019). Circulating essential metals and lung cancer: risk assessment and potential molecular effects. Environ. Int..

[27] Callejon-Leblic B. (2020). Absolute quantification of selenoproteins and selenometabolites in lung cancer human serum by column switching coupled to triple quadrupole inductively coupled plasma mass spectrometry. J. Chromatogr. A.

[28] Juloski J.T. (2020). Colorectal cancer and trace elements alteration. J. Trace Elem. Med. Biol..

[29] Carlisle A.E. (2020). Selenium detoxification is required for cancer-cell survival. Nat. Metabol..

[30] Nunziata C. (2019). Structural analysis of human SEPHS2 protein, a selenocysteine machinery component, over-expressed in triple negative breast cancer. Sci. Rep..

[31] Toyokuni S. (2009). Role of iron in carcinogenesis: cancer as a ferrotoxic disease. Canc. Sci..

[32] Dixon S.J. (2012). Ferroptosis: an iron-dependent form of nonapoptotic cell death. Cell.

[33] Hsing A.W. (1995). Cancer risk following primary hemochromatosis: a population-based cohort study in Denmark. Int. J. Canc..

[34] Jayachandran A. (2020). Association between hereditary hemochromatosis and hepatocellular carcinoma: a comprehensive review. Hepatoma Res..

[35] Quintana Pacheco D.A. (2018). Iron status in relation to cancer risk and mortality: findings from a population-based prospective study. Int. J. Canc..

[36] Muka T. (2017). Dietary mineral intake and lung cancer risk: the Rotterdam Study. Eur. J. Nutr..

[37] Qiao L., Feng Y. (2013). Intakes of heme iron and zinc and colorectal cancer incidence: a meta-analysis of prospective studies. Cancer Causes Control.

[38] Qayyum M.A., Shah M.H. (2014). Comparative study of trace elements in blood, scalp hair and nails of prostate cancer patients in relation to healthy donors. Biol. Trace Elem. Res..

[39] Kuvibidila S.R., Gauthier T., Rayford W. (2004). Serum ferritin levels and transferrin saturation in men with prostate cancer. J. Natl. Med. Assoc..

[40] Sukiennicki G.M. (2019). Iron levels, genes involved in iron metabolism and antioxidative processes and lung cancer incidence. PloS One.

[41] Chen H.F. (2018). A meta-analysis of association between serum iron levels and lung cancer risk. Cell. Mol. Biol..

[42] Phipps O., Brookes M.J., Al-Hassi H.O. (2020). Iron deficiency, immunology, and colorectal cancer. Nutr. Rev..

[43] Cross A.J. (2011). Iron homeostasis and distal colorectal adenoma risk in the prostate, lung, colorectal, and ovarian cancer screening trial. Canc. Prev. Res..

[44] Sohrabi M. (2018). Trace element and heavy metal levels in colorectal cancer: comparison between cancerous and non-cancerous tissues. Biol. Trace Elem. Res..

[45] Marques O. (2014). Iron homeostasis in breast cancer. Canc. Lett..

[46] Shen Y. (2018). Iron metabolism gene expression and prognostic features of hepatocellular carcinoma. J. Cell. Biochem..

[47] Daniels T.R. (2012). The transferrin receptor and the targeted delivery of therapeutic agents against cancer. Biochim. Biophys. Acta.

[48] Duan X. (2018). Tumor associated macrophages deliver iron to tumor cells via Lcn2. Int. J. Physiol. Pathophysiol. Pharmacol..

[49] Doguer C., Ha J.H., Collins J.F. (2018). Intersection of iron and copper metabolism in the mammalian intestine and liver. Comp. Physiol..

[50] Denoyer D. (2015). Targeting copper in cancer therapy: 'Copper that Cancer. Metall.

[51] Stepien M. (2017). Pre-diagnostic copper and zinc biomarkers and colorectal cancer risk in the European Prospective Investigation into Cancer and Nutrition cohort. Carcinogenesis.

[52] Stepien M. (2017). Circulating copper and zinc levels and risk of hepatobiliary cancers in Europeans. Br. J. Canc..

[53] Jouybari L. (2019). Copper concentrations in breast cancer: a systematic review and meta-analysis. Curr. Med. Chem..

[54] Feng Y. (2020). Serum copper and zinc levels in breast cancer: a meta-analysis. J. Trace Elem. Med. Biol..

[55] Kuo H.W. (2002). Serum and tissue trace elements in patients with breast cancer in Taiwan. Biol. Trace Elem. Res..

[56] Geraki K., Farquharson M.J., Bradley D.A. (2002). Concentrations of Fe, Cu and Zn in breast tissue: a synchrotron XRF study. Phys. Med. Biol..

[57] Zhang X., Yang Q. (2018). Association between serum copper levels and lung cancer risk: a meta-analysis. J. Int. Med. Res..

[58] Zablocka-Slowinska K. (2018). Serum and whole blood Zn, Cu and Mn profiles and their relation to redox status in lung cancer patients. J. Trace Elem. Med. Biol..

[59] Aubert L. (2020). Copper bioavailability is a KRAS-specific vulnerability in colorectal cancer. Nat. Commun..

[60] Olechnowicz J. (2018). Zinc status is associated with inflammation, oxidative stress, lipid, and glucose metabolism. J. Physiol. Sci..

[61] Chasapis C.T. (2020). Recent aspects of the effects of zinc on human health. Arch. Toxicol..

[62] Qu X. (2020). Serum zinc levels and multiple health outcomes: implications for zinc-based biomaterials. Bioact Mater..

[63] Wu X., Tang J., Xie M. (2015). Serum and hair zinc levels in breast cancer: a meta-analysis. Sci. Rep..

[64] Jouybari L. (2019). A meta-analysis of zinc levels in breast cancer. J. Trace Elem. Med. Biol..

[65] Riesop D. (2015). Zinc distribution within breast cancer tissue: a possible marker for histological grading?. J. Canc. Res. Clin. Oncol..

[66] Rusch P. (2020). Zinc distribution within breast cancer tissue of different intrinsic subtypes. Arch. Gynecol. Obstet..

[67] Costello L.C., Franklin R.B. (2016). A comprehensive review of the role of zinc in normal prostate function and metabolism; and its implications in prostate cancer. Arch. Biochem. Biophys..

[68] Singh C.K. (2016). Analysis of zinc-exporters expression in prostate cancer. Sci. Rep..

[69] Li D. (2020). Advances of zinc signaling studies in prostate cancer. Int. J. Mol. Sci..

[70] Bialkowska K. (2018). Association of zinc level and polymorphism in MMP-7 gene with prostate cancer in Polish population. PloS One.

[71] Wakwe V.C., Odum E.P., Amadi C. (2019). The impact of plasma zinc status on the severity of prostate cancer disease. Invest. Clin. Urol..

[72] Zhao J. (2016). Comparative study of serum zinc concentrations in benign and malignant prostate disease: a Systematic Review and Meta-Analysis. Sci. Rep..

[73] Wang Y. (2019). Association between serum zinc levels and lung cancer: a meta-analysis of observational studies. World J. Surg. Oncol..

[74] Kolenko V. (2013). Zinc and zinc transporters in prostate carcinogenesis. Nat. Rev. Urol..

[75] Takatani-Nakase T. (2018). Zinc transporters and the progression of breast cancers. Biol. Pharm. Bull..

[76] To P.K. (2020). Growth modulatory role of zinc in prostate cancer and application to cancer therapeutics. Int. J. Mol. Sci..

